# Diagnostic performance of dual-energy CT for differentiating acute intracranial hemorrhage from contrast staining: a systematic review and meta-analysis

**DOI:** 10.3389/fmed.2025.1736860

**Published:** 2026-02-18

**Authors:** Wenbin Ji, Yaosheng Shi

**Affiliations:** Department of Medical Imaging, Shanghai Electric Power Hospital, Shanghai, China

**Keywords:** acute intracranial hemorrhage, contrast staining, diagnostic performance, dual-energy CT, systematic review

## Abstract

**Background:**

Accurate differentiation between acute intracranial hemorrhage (AIH) and contrast staining (CS) on follow-up computed tomography (CT) scans in patients with acute stroke, particularly after endovascular thrombectomy, is a critical clinical challenge. Dual-energy CT (DECT), with its capability for material decomposition, has emerged as a promising solution. This systematic review and meta-analysis aims to determine the overall diagnostic accuracy of DECT for this crucial distinction.

**Methods:**

We conducted a systematic search of PubMed, Embase, the Cochrane Library, and Web of Science from their inception until September 30, 2025. The quality of the included studies was assessed using the Quality Assessment of Diagnostic Accuracy Studies 2 (QUADAS-2) tool. Diagnostic performance was evaluated by calculating pooled estimates for sensitivity, specificity, positive likelihood ratio (PLR), negative likelihood ratio (NLR), diagnostic odds ratio (DOR), and the area under the summary receiver operating characteristic curve (AUC).

**Results:**

Twelve studies, involving 561 patients and 646 lesions, met the eligibility criteria and were included. The pooled diagnostic performance of DECT for differentiating AIH from CS was as follows: sensitivity, 0.90 (95% CI: 0.79–0.96); specificity, 0.98 (95% CI: 0.94–1.00); PLR, 55.91 (95% CI: 14.64–213.53); NLR, 0.10 (95% CI: 0.04–0.23); DOR, 154.76 (95% CI: 64.55–371.02); and AUC, 0.99 (95% CI: 0.97–0.99). Subgroup analysis revealed that in populations with a male proportion ≥60%, DECT demonstrated higher sensitivity, a lower NLR, and a higher AUC. Studies published in or after 2015 showed a significantly higher AUC than those published before 2015. Additionally, the subgroup of patients aged >65 years had a higher AUC compared to younger age groups.

**Conclusion:**

DECT exhibits high diagnostic performance in differentiating AIH from CS. It shows particularly superior results in populations with a higher proportion of males, in older patients, and in more recent studies. However, the presence of potential publication bias may affect the reliability of these findings, underscoring the need for further high-quality studies for validation.

**Systematic review registration:**

https://inplasy.com/wp-content/uploads/2025/10/INPLASY-Protocol-8414.pdf, identifier (INPLASY2025100088).

## Introduction

Acute intracranial hemorrhage (AIH) is a life-threatening neurological emergency encompassing subtypes such as spontaneous intracerebral hemorrhage, traumatic intracranial hemorrhage, and hemorrhage following endovascular treatment. In particular, the accurate identification of AIH versus contrast staining (CS) in patients with acute ischemic stroke undergoing mechanical thrombectomy represents a frequent and decisive diagnostic challenge in modern neurovascular care. It is characterized by high incidence, substantial mortality and disability rates, and a significant potential for causing long-term neurological dysfunction (1, 2). Timely and accurate diagnosis of AIH is therefore critical for guiding urgent clinical management. Failure to identify AIH after endovascular procedures may delay the reversal of anticoagulant/antiplatelet therapy or necessary surgical intervention, potentially resulting in irreversible brain damage or life-threatening cerebral herniation. Conversely, the precise identification of hemorrhage helps to avoid unnecessary or overtreatment (3, 4).

Computed tomography (CT) is the first-line imaging modality for the detection of AIH. On conventional single-energy CT (SECT), acute hemorrhage typically presents as hyperdense areas compared to the surrounding normal brain parenchyma (5). However, a key diagnostic challenge arises in patients who have previously undergone contrast-enhanced CT or endovascular therapy, most notably following mechanical thrombectomy for acute ischemic stroke. In these cases, residual extravasated iodine contrast medium can persist within the brain parenchyma or subarachnoid space, a phenomenon known as “CS.” On SECT images, CS also manifests as hyperdensity, closely mimicking the appearance of AIH and making the two entities difficult to distinguish (6, 7).

The emergence of dual-energy CT (DECT) provides a novel solution to this diagnostic dilemma. Unlike SECT, which utilizes a single X-ray energy spectrum, DECT acquires image data simultaneously at two distinct energy levels (8). By exploiting the differential attenuation characteristics of materials at these two energies, DECT enables material decomposition. The primary component of acute hemorrhage is hemoglobin, a non-iodinated substance, whereas the core component of contrast staining is iodine. Their distinct atomic numbers lead to fundamentally different attenuation patterns under dual-energy scanning (9, 10). Using dedicated post-processing algorithms, DECT can generate material-specific images, such as iodine maps and virtual non-contrast series, which visually isolate and display the distribution of iodine. This capability allows radiologists to differentiate CS from AIH effectively.

A growing number of studies have investigated the value of DECT for differentiating AIH from CS. However, the existing body of evidence is generally constrained by small sample sizes and considerable heterogeneity in scanning protocols, post-processing techniques, and diagnostic criteria. These limitations have contributed to substantial variability in the reported diagnostic performance metrics. This study aims to address this gap by conducting a systematic review and meta-analysis to synthesize the available high-quality evidence, quantitatively pool the diagnostic performance indicators of DECT, and thereby clarify its clinical utility in distinguishing AIH from CS.

## Materials and methods

This diagnostic test accuracy systematic review and meta-analysis was conducted in accordance with the Preferred Reporting Items for Systematic Reviews and Meta-Analyses (PRISMA 2020) statement (11). The study protocol was registered on INPLASY (Registration number: INPLASY2025100088), and the final implementation strictly adhered to the registered protocol without deviations.

### Data sources, search strategy, and selection criteria

A comprehensive literature search was performed in PubMed, Embase, the Cochrane Library, and Web of Science for relevant studies published from database inception to September 30, 2025, without language restrictions. The search strategy was built around the core concepts of “dual-energy CT,” “acute intracranial hemorrhage,” and “contrast staining,” combining both subject headings and free-text terms tailored to the specific syntax of each database. The complete search strategies are available in Supplementary File 1. To ensure comprehensive literature retrieval, we manually screened the reference lists of all included studies and, where applicable, contacted corresponding authors to request any unpublished raw data.

Two investigators independently performed the literature screening. Any discrepancies were resolved by a third investigator, guided by the following eligibility criteria. The inclusion criteria were: (1) Study type: published diagnostic test accuracy studies evaluating both DECT and a reference standard for differentiating AIH from CS; (2) Population: patients with clinically suspected AIH and possible CS. Recognizing distinct clinical contexts, we included studies focusing on two main scenarios: (a) patients after endovascular treatment, most notably mechanical thrombectomy for acute ischemic stroke (where hemorrhage risk is high and differentiation is critical), and (b) patients after a contrast-enhanced CT scan for other indications (where hyperdensity may be incidental). No restrictions were placed on age, sex, or etiology; (3) Index test: use of DECT technology, irrespective of specific scanning parameters or post-processing methods (e.g., iodine maps, virtual non-contrast images); (4) Reference standard: confirmation via follow-up imaging, surgical/pathological findings, or clinical follow-up outcomes; and (5) Outcomes: provision of extractable data for constructing a 2 × 2 diagnostic contingency table (true positives, false positives, true negatives, false negatives) or directly reported sensitivity, specificity, positive likelihood ratio (PLR), negative likelihood ratio (NLR), or area under the receiver operating characteristic curve (AUC). Exclusion criteria included non-diagnostic studies, lack of verification by a reference standard, and incomplete or inaccessible data.

### Data collection and quality assessment

Two investigators independently extracted data using a standardized Excel form. The extracted information included: (1) baseline study characteristics (first author, publication year, country/region, study design); (2) patient and lesion characteristics (total sample size, age, sex distribution, number of lesions, time interval to reference standard, primary clinical context); and (3) details of the reference standard modality and diagnostic performance metrics. Disagreements during data extraction were resolved by joint re-examination of the original articles and discussion. If consensus could not be reached, a third investigator made the final determination.

The methodological quality and risk of bias of the included studies were assessed independently by two investigators using the Quality Assessment of Diagnostic Accuracy Studies 2 (QUADAS-2) tool (12). This tool evaluates four domains: patient selection, index test, reference standard, and flow and timing. Each domain was rated as “low,” “high,” or “unclear” risk of bias. Disagreements were resolved using the same consensus process as for data extraction.

### Statistical analysis

The pooled sensitivity and specificity with their 95% confidence intervals (CIs) were calculated and visualized using forest plots. The combined PLR and NLR with their 95% CIs were also computed; a PLR > 10 or NLR < 0.1 was considered to indicate high diagnostic value. A summary receiver operating characteristic (SROC) curve was plotted, and the pooled area under the curve (AUC) was calculated. All pooled estimates were derived using a bivariate generalized linear mixed model with random effects (13, 14). Heterogeneity across studies was assessed using the *Q*-test and *I^2^* statistic, with a *p*-value ≤ 0.10 or *I^2^* ≥ 50% indicating substantial heterogeneity (15, 16). To explore potential sources of heterogeneity, pre-specified subgroup analyses were conducted based on publication year, country, study design, patient age, and sex ratio. Publication bias was evaluated using Deeks’ funnel plot asymmetry test, with a *p*-value ≤ 0.05 suggesting potential bias (17). All statistical analyses were performed using Stata version 18.0, with a two-sided significance level of *α* = 0.05.

## Results

### Literature search

**Figure 1:**
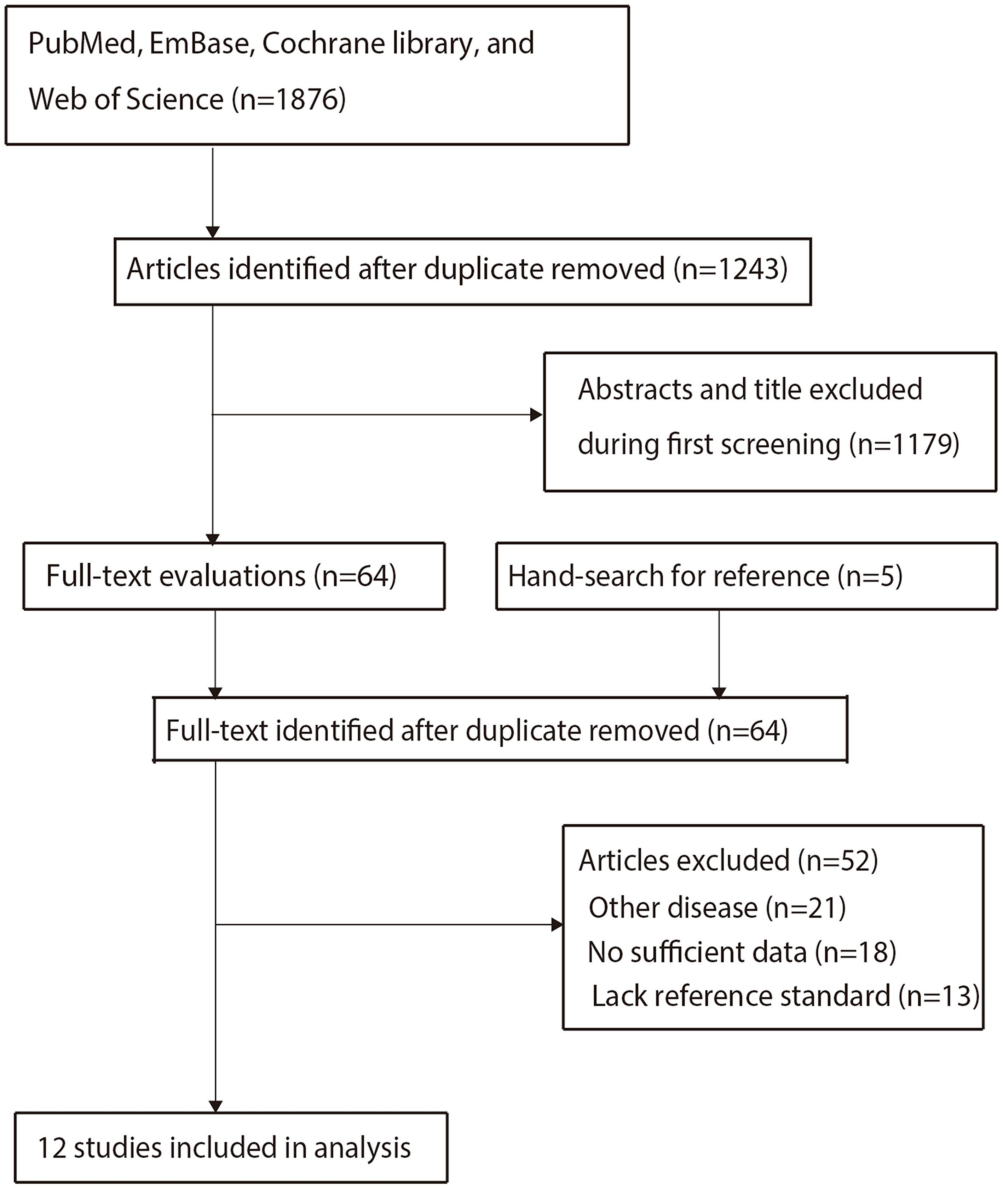
Flowchart of literature search and selection.

The initial search identified 1,876 potentially relevant records. After removing duplicates, 1,243 unique records remained. Screening of titles and abstracts led to the exclusion of 1,179 studies that were non-diagnostic, involved ineligible patient populations, or were otherwise irrelevant. The full texts of the remaining 64 articles were assessed for eligibility. Of these, 52 were excluded. A detailed list of these excluded studies, grouped by the primary reason for exclusion, is provided in Supplementary File 2. The main reasons for exclusion were incomplete data, lack of reference standard verification, or inclusion of other disease conditions. Ultimately, 12 diagnostic test accuracy studies, comprising 561 patients and 646 lesions, were included in the meta-analysis (18–29). The literature selection process is detailed in Figure 1.

### Study characteristics

Among the 12 included studies, 10 were retrospective cohort studies and 2 were prospective cohort studies. Geographically, 2 studies were from Asia and 10 were from Europe or North America. The mean or median age of patients ranged from 55.0 to 73.4 years. As detailed in Table 1, the primary clinical context for the majority of included studies (8 out of 12) was the assessment of patients after endovascular treatment, predominantly mechanical thrombectomy. Four studies evaluated populations after contrast-enhanced CT for other indications. The primary DECT post-processing techniques employed were iodine maps and virtual non-contrast images. All included studies used follow-up imaging as the reference standard. To address potential variability in the reference standard, we extracted detailed information on the specific imaging modality used and the reported time interval between the index DECT and the reference assessment. These data are presented in Table 1. The reference modalities included follow-up CT, MRI, or a combination thereof, and the time intervals varied across studies, with the majority performing follow-up within 72 h. Quality assessment using the QUADAS-2 tool indicated that the overall quality of the included studies was acceptable. However, a high risk of bias was identified primarily in the ‘patient selection’ domain, largely due to insufficient description of the enrollment methods. The specific results of the quality assessment are summarized in Table 2.

**Table 1:** The baseline characteristics of included studies and involved patients.

Study	Country	Study design	Sample size	Age (years)	Male (%)	No of lesions	Time interval to reference standard	Primary clinical context	Reference modality	True positive	False positive	False negative	True negative
Gupta 2010 (18)	USA	Retrospective	11	67.0	55.6	28	24–48 h	Post-contrast material	Unenhanved CT or MRI	6	0	2	20
Phan 2012 (19)	USA	Retrospective	40	64.9	67.5	147	24–48 h	Post-contrast material	Unenhanved CT or MRI	72	0	5	70
Morhard 2014 (20)	Germany	Retrospective	60	73.4	48.3	48	24–144 h	Post-endovascular therapy	Unenhanved CT or MRI	5	0	0	43
Tijssen 2014 (21)	Netherlands	Retrospective	22	56.0	50.0	19	24 h	Post-endovascular therapy	Follow-up CT	1	2	0	16
Watanabe 2014 (22)	Japan	Retrospective	36	60.0	66.7	40	NA	Post-contrast material	Contrast enhancement CT	22	1	1	16
Bodanapally 2017 (23)	USA	Retrospective	48	69.0	69.9	48	< 72 h	Post-contrast material	Follow-up CT	22	2	0	24
Bonatti 2018 (24)	Italy	Retrospective	85	70.0	61.2	85	24 h	Post-endovascular therapy	Follow-up CT	5	9	0	71
Zaouak 2020 (25)	Belgium	Prospective	35	55.0	17.1	35	24–48 h	Post-endovascular therapy	Follow-up CT	2	0	1	32
Wang 2021 (26)	China	Prospective	44	66.3	63.6	44	24 h	Post-endovascular therapy	Follow-up CT	19	0	2	23
Grkovski 2023 (27)	Switzerland	Retrospective	39	69.0	53.8	18	48–72 h	Post-endovascular therapy	Unenhanved CT or MRI	9	0	1	8
Pacielli 2024 (28)	Italy	Retrospective	44	69.0	50.0	37	24 h	Post-endovascular therapy	Follow-up CT	19	1	8	9
Pressram 2025 (29)	USA	Retrospective	97	66.0	45.4	97	24 h	Post-endovascular therapy	Follow-up MRI	25	0	16	66

**Table 2:** Risk of bias and applicability using the QUADAS-2 tool.

Study	Risk of bias				Applicability		
	Patient selection	Index test	Reference standard	Flow and timing	Patient selection	Index test	Reference standard
Gupta 2010 (18)	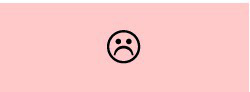			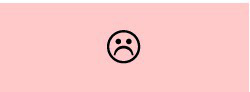			
Phan 2012 (19)	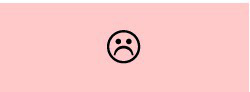			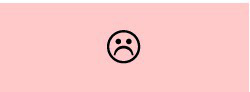			
Morhard 2014 (20)	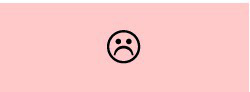						
Tijssen 2014 (21)	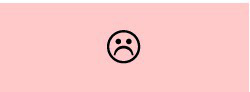						
Watanabe 2014 (22)	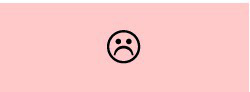	?					
Bodanapally 2017 (23)	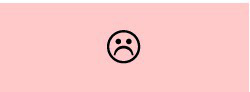						
Bonatti 2018 (24)	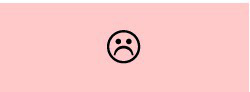	?					
Zaouak 2020 (25)	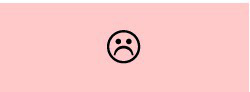						
Wang 2021 (26)	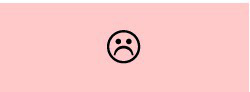			?			
Grkovski 2023 (27)	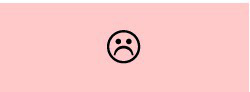						
Pacielli 2024 (28)	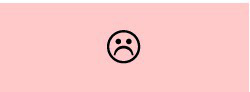						
Pressram 2025 (29)	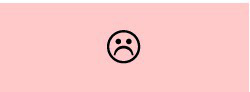						

### Sensitivity and specificity

**Figure 2:**
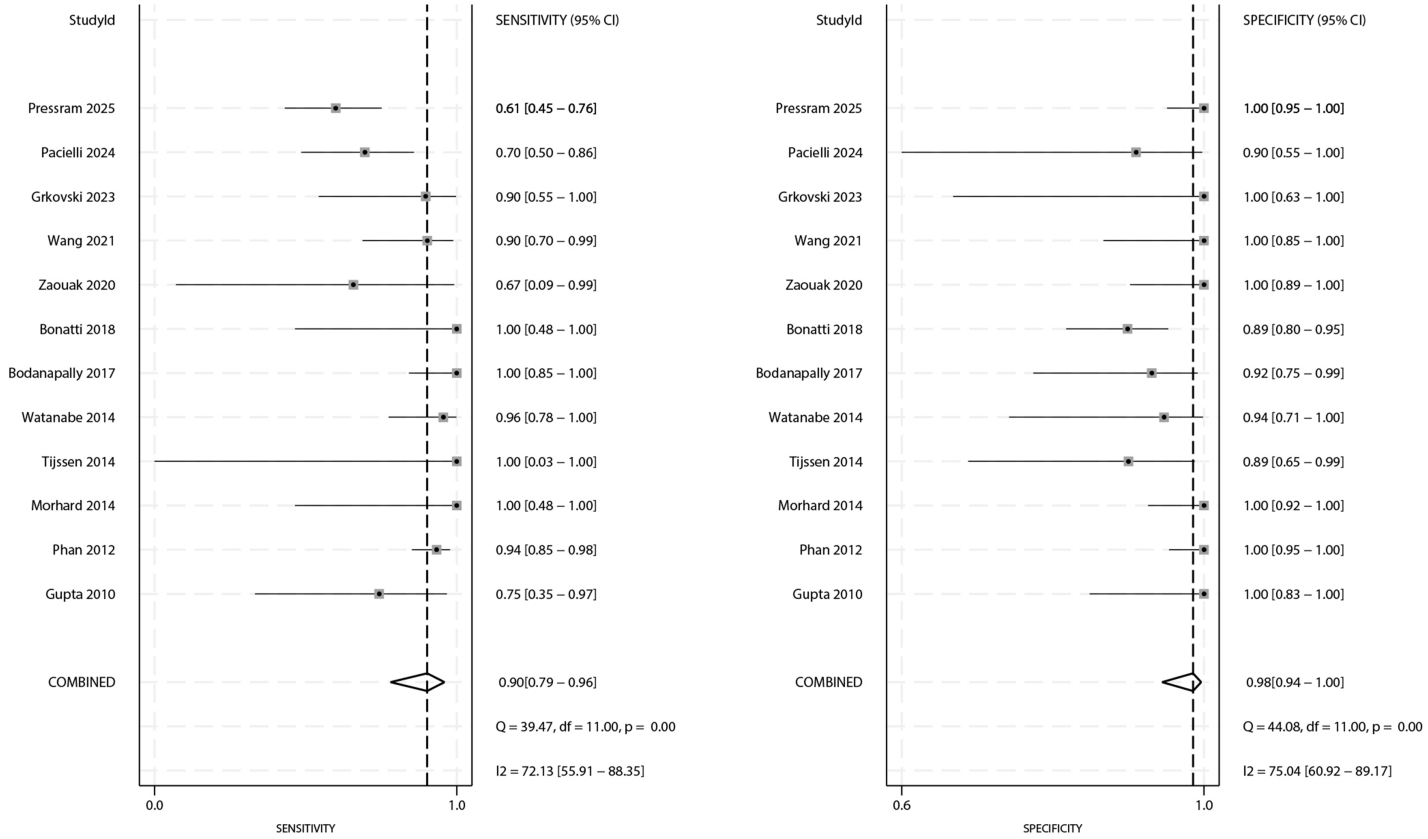
Forest plot of pooled sensitivity and specificity of DECT for differentiating AIH from CS.

The forest plots for sensitivity and specificity are shown in Figure 2. The pooled sensitivity was 0.90 (95% CI: 0.79–0.96), and the pooled specificity was 0.98 (95% CI: 0.94–1.00). Significant heterogeneity was observed for both sensitivity (*I^2^* = 72.13%; *p* < 0.01) and specificity (*I^2^* = 75.04%; *p* < 0.01). Subgroup analysis indicated that sensitivity was significantly higher in populations with a male proportion ≥60% compared to those with a male proportion <60% (ratio: 1.34; 95% CI: 1.15–1.56). No other subgroups showed statistically significant differences in sensitivity (Table 3).

**Table 3:** Subgroup analysis for diagnostic performance.

Diagnostic metrics	Factors	Subgroups	ES and 95% CI	*I^2^* (%)	Difference between subgroups
Sensitivity	Publication year	Before 2015	0.93 (0.86–0.97)	14.91	1.06 (0.87–1.28)
		2015 or after	0.88 (0.67–0.96)	71.30	
	Country	Europe or USA	0.89 (0.73–0.96)	71.83	0.96 (0.82–1.11)
		Asia	0.93 (0.86–0.97)	0.00	
	Study design	Prospective	0.88 (0.75–0.94)	0.00	0.96 (0.81–1.12)
		Retrospective	0.92 (0.77–0.97)	75.65	
	Age (years)	≥ 65.0	0.88 (0.70–0.96)	72.17	0.94 (0.79–1.11)
		< 65.0	0.94 (0.85–0.97)	18.23	
	Male (%)	≥ 60.0	0.95 (0.89–0.98)	0.00	1.34 (1.15–1.56)
		< 60.0	0.71 (0.60–0.80)	3.69	
Specificity	Publication year	Before 2015	0.99 (0.88–1.00)	69.16	1.02 (0.95–1.10)
		2015 or after	0.97 (0.91–0.99)	73.17	
	Country	Europe or USA	0.99 (0.93–1.00)	77.50	1.02 (0.97–1.08)
		Asia	0.97 (0.91–0.99)	0.00	
	Study design	Prospective	0.99 (0.94–1.00)	0.00	1.01 (0.96–1.06)
		Retrospective	0.98 (0.92–0.99)	74.04	
	Age (years)	≥ 65.0	0.98 (0.91–1.00)	76.74	1.00 (0.92–1.09)
		< 65.0	0.98 (0.87–1.00)	70.16	
	Male (%)	≥ 60.0	0.96 (0.88–0.98)	56.26	0.97 (0.89–1.05)
		< 60.0	0.99 (0.88–1.00)	67.46	
PLR	Publication year	Before 2015	89.11 (6.99–1136.55)	32.58	2.56 (0.15–44.57)
		2015 or after	34.79 (9.52–127.17)	31.99	
	Country	Europe or USA	61.76 (11.96–318.98)	54.46	1.66 (0.20–14.06)
		Asia	37.27 (9.47–146.63)	0.00	
	Study design	Prospective	97.12 (13.76–685.43)	0.00	2.39 (0.24–23.77)
		Retrospective	40.58 (12.16–135.39)	49.21	
	Age (years)	≥ 65.0	48.53 (9.38–251.14)	47.63	0.83 (0.05–13.02)
		< 65.0	58.19 (6.43–526.49)	34.79	
	Male (%)	≥ 60.0	21.30 (7.57–59.94)	0.40	0.23 (0.01–4.91)
		< 60.0	96.36 (5.34–1739.08)	17.65	
NLR	Publication year	Before 2015	0.07 (0.03–0.15)	50.52	0.54 (0.14–2.13)
		2015 or after	0.13 (0.04–0.37)	70.06	
	Country	Europe or USA	0.11 (0.04–0.29)	73.22	1.57 (0.44–5.63)
		Asia	0.07 (0.03–0.15)	0.00	
	Study design	Prospective	0.13 (0.06–0.27)	32.90	1.62 (0.44–5.96)
		Retrospective	0.08 (0.03–0.25)	77.48	
	Age (years)	≥ 65.0	0.12 (0.04–0.33)	72.91	2.00 (0.53–7.54)
		< 65.0	0.06 (0.03–0.15)	64.90	
	Male (%)	≥ 60.0	0.05 (0.02–0.12)	0.00	0.17 (0.07–0.45)
		< 60.0	0.29 (0.20–0.41)	0.00	
DOR	Publication year	Before 2015	281.77 (60.11–1320.91)	13.20	2.62 (0.39–17.48)
		2015 or after	107.59 (35.69–324.28)	0.00	
	Country	Europe or USA	129.61 (49.52–339.26)	0.00	0.36 (0.06–2.11)
		Asia	358.61 (81.53–1577.37)	0.00	
	Study design	Prospective	179.76 (36.67–881.34)	0.00	1.22 (0.19–7.79)
		Retrospective	146.82 (56.94–378.56)	0.40	
	Age (years)	≥ 65.0	124.84 (43.49–358.36)	0.00	0.54 (0.06–4.53)
		< 65.0	232.11 (36.46–1477.73)	27.90	
	Male (%)	≥ 60.0	339.84 (95.66–1207.33)	0.00	4.43 (0.80–24.71)
		< 60.0	76.63 (24.04–244.20)	0.00	
AUC	Publication year	Before 2015	0.95 (0.93–0.97)	-	0.97 (0.95–0.99)
		2015 or after	0.98 (0.97–0.99)	-	
	Country	Europe or USA	0.99 (0.97–0.99)	-	1.00 (0.98–1.01)
		Asia	0.99 (0.98–1.00)	-	
	Study design	Prospective	0.99 (0.98–1.00)	-	1.00 (0.99–1.01)
		Retrospective	0.99 (0.98–1.00)	-	
	Age (years)	≥ 65.0	0.99 (0.97–0.99)	-	1.03 (1.00–1.05)
		< 65.0	0.96 (0.93–0.97)	-	
	Male (%)	≥ 60.0	0.98 (0.96–0.99)	-	1.24 (1.18–1.30)
		< 60.0	0.79 (0.75–0.82)	-	

### PLR and NLR

**Figure 3:**
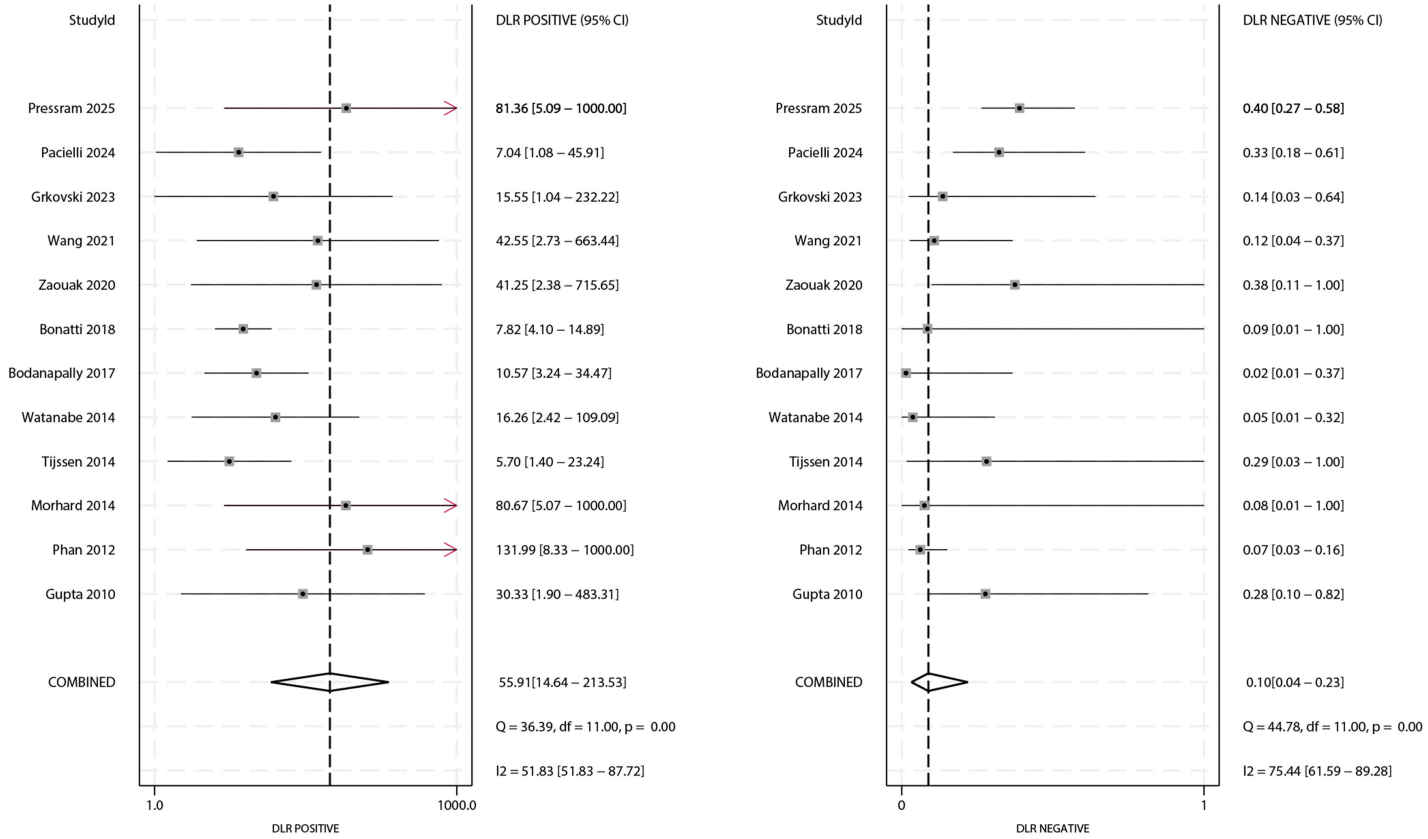
Forest plot of pooled positive likelihood ratio (PLR) and negative likelihood ratio (NLR) of DECT for differentiating AIH from CS.

The forest plots for PLR and NLR are presented in Figure 3. The pooled PLR was 55.91 (95% CI: 14.64–213.53), and the pooled NLR was 0.10 (95% CI: 0.04–0.23). Significant heterogeneity was observed for both PLR (*I^2^* = 51.83%; *p* < 0.01) and NLR (*I^2^* = 75.44%; *p* < 0.01). Subgroup analysis showed that the NLR was significantly lower in populations with a male proportion ≥60% (ratio: 0.17; 95% CI: 0.07–0.45; Table 3).

### DOR

**Figure 4:**
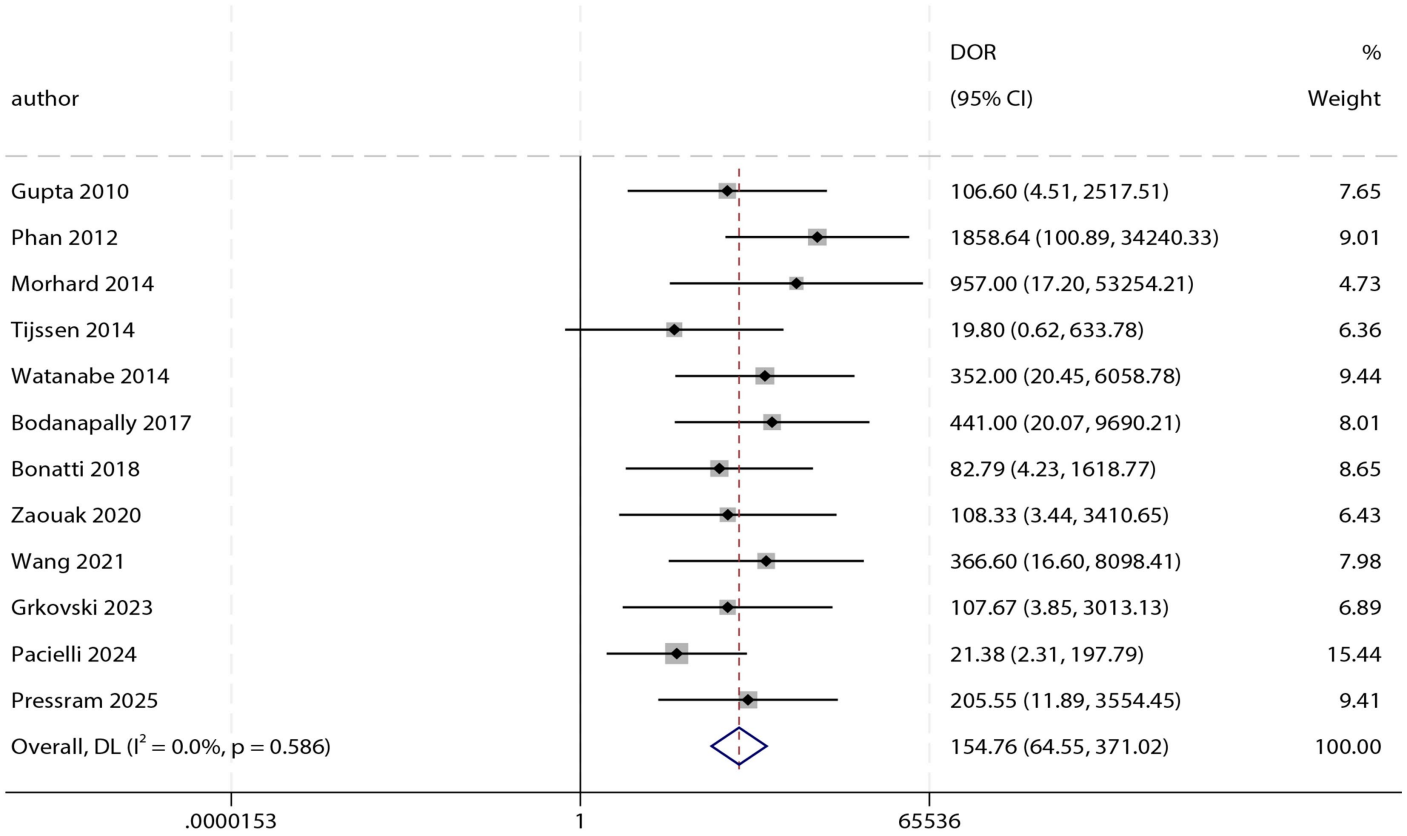
Forest plot of pooled diagnostic odds ratio (DOR) of DECT for differentiating AIH from CS.

The pooled diagnostic odds ratio (DOR) is shown in Figure 4. The combined DOR was 154.76 (95% CI: 64.55–371.02; *p* < 0.001), with no significant heterogeneity observed across studies (*I^2^* = 0.0%; *p* = 0.586). Subgroup analysis revealed no statistically significant differences in DOR between any of the analyzed subgroups (Table 3).

### AUC

**Figure 5:**
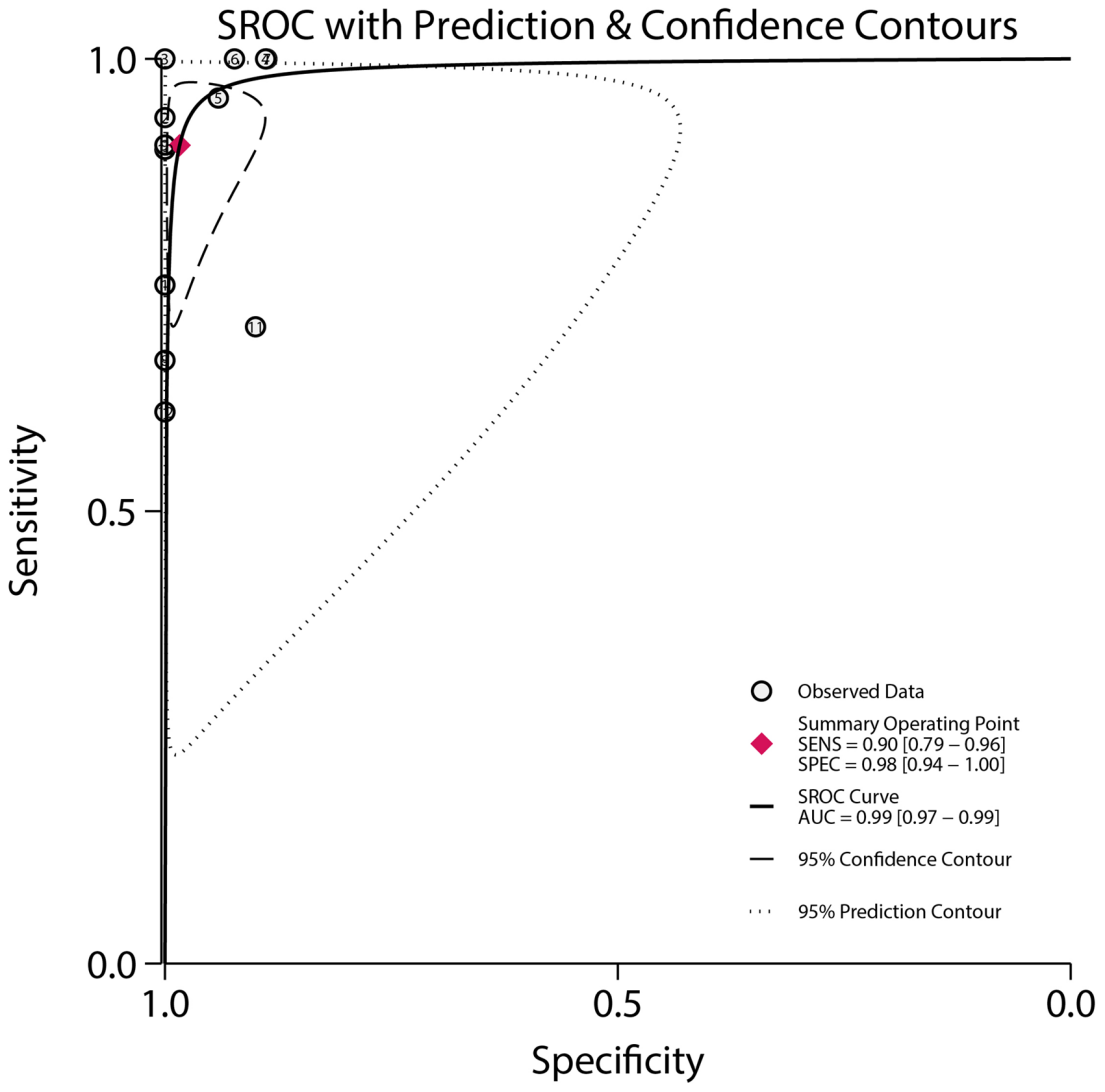
Summary receiver operating characteristic (SROC) curve of DECT for differentiating AIH from CS.

The SROC curve and the AUC are shown in Figure 5. The pooled AUC was 0.99 (95% CI: 0.97–0.99). Subgroup analysis revealed that studies published in or after 2015 had a significantly higher AUC than those published before 2015 (ratio: 1.03; 95% CI: 1.01–1.05). Additionally, significantly higher AUC values were observed in subgroups with age >65 years (ratio: 1.03; 95% CI: 1.00–1.05) and male proportion ≥60% (ratio: 1.24; 95% CI: 1.18–1.30) compared to their respective reference subgroups (Table 3).

### Publication bias

**Figure 6:**
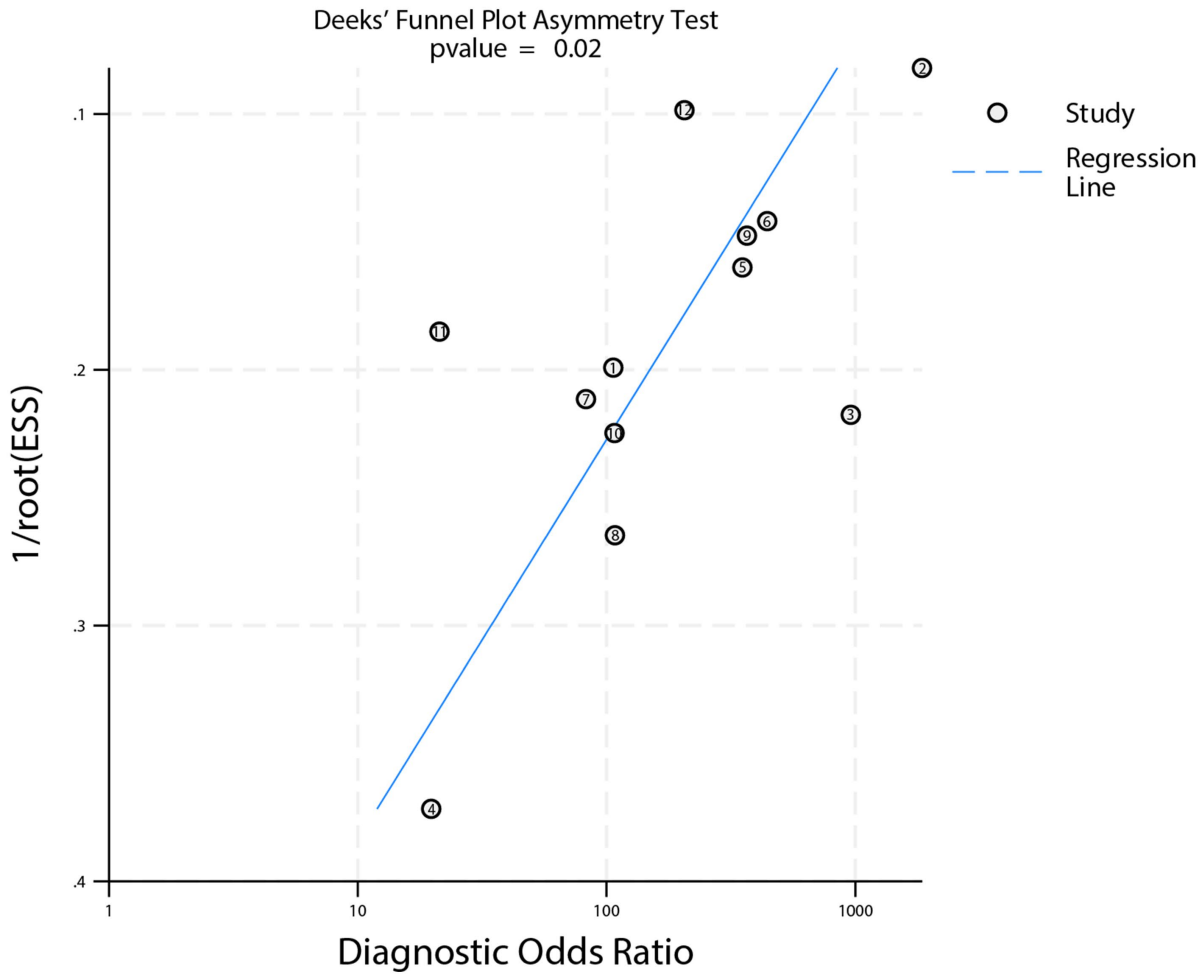
Deeks’ funnel plot for assessing publication bias of studies on DECT differentiating AIH from CS.

Deeks’ funnel plot asymmetry test for publication bias is shown in Figure 6. The test indicated the presence of significant publication bias (*p* = 0.02).

## Discussion

Timely and accurate diagnosis of AIH is crucial for guiding clinical intervention and improving patient outcomes. However, conventional SECT has long posed a diagnostic challenge in distinguishing CS from AIH in clinical practice, a dilemma that is now commonplace in the follow-up imaging of patients treated with mechanical thrombectomy for acute ischemic stroke. Through a systematic review and meta-analysis of 12 studies, this research provides, for the first time with a substantial pooled sample size, a comprehensive quantitative assessment of the diagnostic performance of DECT in differentiating AIH from CS, thereby offering an evidence-based foundation for its application in neuroemergency imaging. The results demonstrate that DECT achieves a pooled sensitivity of 0.90 (95% CI: 0.79–0.96), a pooled specificity of 0.98 (95% CI: 0.94–1.00), and an AUC of 0.99, indicating its strong discriminative capability.

The high specificity (0.98) indicates that DECT rarely misclassifies CS as AIH (low false-positive rate), effectively avoiding unnecessary reversal of antiplatelet therapy or surgical intervention. This is particularly critical for patients post-endovascular treatment, especially following mechanical thrombectomy, who require a careful balance between bleeding risk and antithrombotic needs, as it helps mitigate the risk of thrombotic or ischemic events that could result from the premature discontinuation of necessary antithrombotic therapy due to a false-positive hemorrhage diagnosis (3, 4). It is noteworthy that the pooled results are predominantly driven by studies in the post-thrombectomy population (8 out of 12 studies), which is the clinical setting where this diagnostic dilemma is most acute and consequential. While DECT is also technically applicable to the less common scenario of hyperdensity after routine contrast CT, its paramount clinical value lies in guiding urgent management decisions following thrombectomy. Conversely, the high sensitivity (0.90) suggests a low probability of DECT missing true AIH, enabling timely identification of cases requiring urgent intervention and preventing irreversible complications such as cerebral herniation due to delayed diagnosis. The pooled positive likelihood ratio (PLR > 10) and negative likelihood ratio (NLR < 0.1) further validate the clinical utility of DECT: a positive DECT result significantly increases the probability of true AIH, while a negative result can effectively rule it out. Together, these metrics provide robust evidence-based support for clinical decision-making (30).

The foundation of this high diagnostic performance lies primarily in the material-specific discrimination capability of DECT. By leveraging dual-energy spectral data to capture the differential X-ray attenuation properties between iodine (the core component of contrast agents) and hemoglobin (the main component of AIH), and utilizing post-processing techniques such as iodine maps and virtual non-contrast images, DECT enables clear and intuitive differentiation between CS and AIH (9, 10).

Subgroup analyses revealed population-specific variations in the diagnostic performance of DECT. In populations with a male proportion ≥60%, DECT demonstrated significantly higher sensitivity, a higher AUC, and a lower NLR. Males represent a high-risk group for vascular diseases, and their AIH is often spontaneous or post-procedural, potentially characterized by larger lesion volumes and higher hemoglobin concentrations, which may lead to more distinct attenuation differences from iodine-based CS (31). Additionally, CS in male patients might present with more stable residual contrast agent after endovascular treatment, potentially reducing diagnostic ambiguity. Studies published in or after 2015 showed a significantly higher AUC than those published earlier, which aligns with continuous technological advancements in DECT. Since 2015, improvements in spectral resolution and post-processing algorithms have likely enabled more precise differentiation between iodine and hemoglobin attenuation patterns (32). Concurrently, increased clinical experience and the gradual standardization of scanning protocols and diagnostic criteria may have further enhanced diagnostic accuracy. Furthermore, the subgroup of patients aged >65 years exhibited a higher AUC than younger age groups. This could be attributed to the fact that AIH in older patients often occurs against a background of cerebral atrophy or leukoaraiosis, potentially enhancing lesion-to-brain tissue contrast. Moreover, CS in older patients might tend to be more localized after endovascular treatment, making it easier to distinguish from the typically more diffuse pattern of AIH (33).

Previous investigations on the use of DECT to differentiate AIH from CS have predominantly been small-scale, single-center explorations with notable limitations. First, sample sizes were often fewer than 50 cases, leading to unstable estimates of diagnostic performance (18, 19, 21–23, 25–28). Second, significant heterogeneity existed in scanning parameters, post-processing methods, and diagnostic criteria, resulting in considerable variability in reported outcomes. The present meta-analysis overcomes these limitations through a large-scale synthesis, rigorous quality assessment, detailed exploration of heterogeneity, and comprehensive evaluation of multiple diagnostic metrics. Compared to recently published reviews (34), this study features a more updated search timeframe, includes a greater number of primary studies, and provides more in-depth exploratory analyses.

This study has several limitations. First, a high risk of bias was identified in the “patient selection” domain of the included studies, which may limit the representativeness of the enrolled populations and affect the generalizability of the results. Second, Deeks’ funnel plot asymmetry test indicated significant publication bias, suggesting that the pooled diagnostic performance of DECT might be overestimated due to the under-publication of studies with negative or less favorable results. Third, the preponderance of retrospective studies (10 out of 12) introduces a potential for selection bias, whereas prospective designs are generally considered more robust. Fourth, although subgroup analyses explored several clinical and methodological factors, they did not assess the potential impact of different DECT scanner models or specific post-processing parameters on diagnostic performance, potentially overlooking the influence of key technical variables. Fifth, an important limitation concerns the variability in the reference standard used across studies. While all studies employed follow-up imaging for verification, the specific modality and the time interval between the index DECT and reference imaging were not standardized. For instance, a rapidly resolving contrast stain or a small hemorrhage might be missed on delayed imaging, potentially leading to verification bias. Furthermore, the diagnostic performance of the reference tests themselves is not perfect. This heterogeneity in the reference standard introduces a layer of uncertainty into our pooled estimates and precluded a meaningful subgroup analysis based on these factors. Future prospective studies should aim to define and adhere to a consistent, optimal reference standard protocol. Finally, most included studies were conducted in Europe and North America, with limited data from Africa, South America, or other regions, which may introduce geographical bias and limit the global applicability of the findings.

## Conclusion

This systematic review and meta-analysis suggests that DECT demonstrates excellent diagnostic performance in differentiating acute intracranial hemorrhage from contrast staining, particularly in populations with a higher male proportion, individuals over 65 years of age, and in studies conducted after 2015. These findings provide robust evidence supporting the potential of DECT to guide critical clinical decision-making in neurovascular emergencies. However, the interpretation of these results should consider the noted limitations, including the risk of bias in patient selection and potential publication bias, which may affect the generalizability of the findings. Despite these caveats, the accumulated evidence indicates that DECT holds significant value for clinical adoption. Future high-quality, multi-center prospective studies are warranted to further validate its diagnostic efficacy, promote technical standardization and individualized application, and ultimately improve diagnostic accuracy and therapeutic outcomes for patients with AIH.

## Data Availability

The original contributions presented in the study are included in the article/Supplementary material, further inquiries can be directed to the corresponding author.
