# Supplementary material for: Diagnostic performance of dual-energy CT for differentiating acute intracranial hemorrhage from contrast staining: a systematic review and meta-analysis
Source: Front Med (Lausanne). 2026 Feb 18;12:1736860. doi: 10.3389/fmed.2025.1736860 (PMC12957155; doi:10.3389/fmed.2025.1736860)
Supplement: Supplementary file 1 [file Table_1.docx]

**PubMed:**

#1 ((((dual energy[Title/Abstract]) OR (dual source[Title/Abstract])) OR (multi energy[Title/Abstract])) OR (multi source[Title/Abstract])) OR (spectral CT[Title/Abstract])

#2 (Tomography, X-Ray Computed[MeSH Terms]) OR (computed tomography[Title/Abstract]) OR (CT[Title/Abstract])

#3 #1 AND #2

#4 (Intracranial Hemorrhages[MeSH Terms]) OR (intracranial hemorrhage[Title/Abstract]) OR (intracerebral hemorrhage[Title/Abstract]) OR (subarachnoid hemorrhage[Title/Abstract])

#5 (brain[Title/Abstract]) OR (cerebral[Title/Abstract]) OR (intracranial[Title/Abstract])

#6 (hemorrhage[Title/Abstract]) OR (haemorrhage[Title/Abstract]) OR (bleed[Title/Abstract]) OR (OR ("contrast staining"[Title/Abstract]) OR ("contrast extravasation"[Title/Abstract])

#7 #5 AND #6

#8 #4 OR #7

#9 (((("diagnostic accuracy"[Title/Abstract]) OR (sensitivity[Title/Abstract])) OR (specificity[Title/Abstract])) OR ("ROC curve"[Title/Abstract])) OR ("area under the curve"[Title/Abstract])

#10 (Sensitivity and Specificity[MeSH Terms]) OR (ROC Curve[MeSH Terms])

#11 #9 OR #10

#12 #3 AND #8 AND #11

**EmBase:**

1. (dual energy or dual source or multi energy or multi source or spectral ct).ab,ti.

2. exp *dual energy computed tomography/ or exp *computer assisted tomography/

3. 1 or 2

4. (brain or cerebral or intracranial or intracerebral).ab,ti.

5. exp *brain/ or exp *intracranial hemorrhage/

6. 4 or 5

7. (hemorrhage or haemorrhage or bleed or differentiat* or distinguis* or "contrast staining" or calcification).ab,ti.

8. exp *brain hemorrhage/

9. 7 or 8

10. ("diagnostic accuracy" or sensitivity or specificity or "roc curve" or "area under the curve" or "receiver operating characteristic").ab,ti.

11. exp *"sensitivity and specificity"/ or exp *"receiver operating characteristic"/

12. 10 or 11

13. 3 and 6 and 9 and 12

**Cochrane Library:**

#1 MeSH descriptor: [Tomography, X-Ray Computed] explode all trees

#2 (dual energy OR dual source OR multi energy OR multi source OR "spectral CT")::ti,ab,kw

#3 #1 OR #2

#4 MeSH descriptor: [Intracranial Hemorrhages] explode all trees

#5 MeSH descriptor: [Brain] explode all trees

#6 (brain OR cerebral OR intracranial OR intracerebral)::ti,ab,kw

#7 #4 OR #5 OR #6

#8 MeSH descriptor: [Hemorrhage] explode all trees

#9 (hemorrhage OR haemorrhage OR bleed OR differentiat* OR distinguis* OR "contrast staining" OR calcification)::ti,ab,kw

#10 #8 OR #9

#11 MeSH descriptor: [Sensitivity and Specificity] explode all trees

#12 MeSH descriptor: [ROC Curve] explode all trees

#13 ("diagnostic accuracy" OR sensitivity OR specificity OR "ROC curve" OR "area under the curve")::ti,ab,kw

#14 #11 OR #12 OR #13

#15 #3 AND #7 AND #10 AND #14

**Web of Science:**

#1 TS=((dual energy OR dual source OR multi energy OR multi source OR "spectral CT") AND (computed tomography OR CT))

#2 TS=(brain OR cerebral OR intracranial OR intracerebral)

#3 TS=((hemorrhage OR haemorrhage OR bleed) AND (differentiat* OR distinguis* OR "contrast staining"))

#4 TS=("diagnostic accuracy" OR sensitivity OR specificity OR "ROC curve" OR "area under the curve" OR "receiver operating characteristic")

#5 #1 AND #2 AND #3 AND #4
