# Supplementary material for: Diagnostic performance of dual-energy CT for differentiating acute intracranial hemorrhage from contrast staining: a systematic review and meta-analysis
Source: Front Med (Lausanne). 2026 Feb 18;12:1736860. doi: 10.3389/fmed.2025.1736860 (PMC12957155; doi:10.3389/fmed.2025.1736860)
Supplement: Supplementary file 2 [file Table_2.docx]

**Incomplete data (n=18):**

1. Hatim AH, Kabrah DK, Hassan ME, Qabha HM. Contrast Extravasation on Computed Tomography Angiography as a Predictor of Hematoma Expansion and Outcomes in Spontaneous Intracerebral Hemorrhage: A Retrospective Study. Cureus. 2025 Oct 31;17(10):e95853. doi: 10.7759/cureus.95853.
2. Jiang XY, Zhang SH, Xie QZ, Yin ZJ, Liu QY, Zhao MD, Li XL, Mao XJ. Evaluation of Virtual Noncontrast Images Obtained from Dual-Energy CTA for Diagnosing Subarachnoid Hemorrhage. AJNR Am J Neuroradiol. 2015 May;36(5):855-60. doi: 10.3174/ajnr.A4223.
3. Kim SJ, Lim HK, Lee HY, Choi CG, Lee DH, Suh DC, Kim SM, Kim JK, Krauss B. Dual-energy CT in the evaluation of intracerebral hemorrhage of unknown origin: differentiation between tumor bleeding and pure hemorrhage. AJNR Am J Neuroradiol. 2012 May;33(5):865-72. doi: 10.3174/ajnr.A2890.
4. Sharma S, Toppo SK, Ranjan RK, Xalxo AR, Rai N, Sharma MK, Kumar A. Role of MDCT Angiography in Evaluation of Nontraumatic Subarachnoid Hemorrhage. J Pharm Bioallied Sci. 2025 Jun;17(Suppl 2):S1350-S1352. doi: 10.4103/jpbs.jpbs_98_25.
5. Park BS, Han JH, Yoon WK, Suh SI. Cerebral Contrast Staining on Dual-Energy Computed Tomography After Coil Embolization of Unruptured Intracranial Aneurysms: Frequency, Risk Factors, and Clinical Implications. World Neurosurg. 2025 May;197:123930. doi: 10.1016/j.wneu.2025.123930.
6. Ogata A, Ogasawara K, Nishihara M, Takamori A, Furukawa T, Ide T, Ito H, Yoshioka F, Nakahara Y, Masuoka J, Koike H, Irie H, Abe T. Subarachnoid iodine leakage on dual-energy computed tomography after mechanical thrombectomy is associated with malignant brain edema. J Neurointerv Surg. 2025 Feb 14;17(3):248-253. doi: 10.1136/jnis-2023-021413.
7. An K, Chen C, Dong M, Chen W, Cao Y. CT Texture-Based Nomogram in Ischemic Stroke to Differentiate Intracerebral Hemorrhage from Contrast Extravasation after Thrombectomy. Cerebrovasc Dis. 2024;53(4):457-466. doi: 10.1159/000536667.
8. Gong C, Wang Y, Yuan J, Zhang J, Jiang S, Xu T, Chen Y. The Association of the Spatial Location of Contrast Extravasation with Symptomatic Intracranial Hemorrhage after Endovascular Therapy in Acute Ischemic Stroke Patients. Curr Neurovasc Res. 2023;20(3):354-361. doi: 10.2174/1567202620666230721101413.
9. DiNitto J, Feldman M, Grimaudo H, Mummareddy N, Ahn S, Bhamidipati A, Anderson D, Ramirez-Giraldo JC, Fusco M, Chitale R, Froehler MT. Flat-panel dual-energy head computed tomography in the angiography suite after thrombectomy for acute stroke: A clinical feasibility study. Interv Neuroradiol. 2023 Feb 14:15910199231157462. doi: 10.1177/15910199231157462.
10. Chrzan R, Łasocha B, Brzegowy P, Popiela T. Dual energy computed tomography in differentiation of iodine contrast agent staining from secondary brain haemorrhage in patients with ischaemic stroke treated with thrombectomy. Neurol Neurochir Pol. 2022;56(1):68-74. doi: 10.5603/PJNNS.a2022.0005.
11. Pan XH, Qiu K, Zhu FY, Shi HB, Liu S. Immediate postinterventional flat-panel CT: Differentiation of hemorrhagic transformation from contrast exudation of acute ischemic stroke patients after thrombectomy. Acta Radiol. 2023 Apr;64(4):1600-1607. doi: 10.1177/02841851221122429.
12. Ma C, Xu D, Hui Q, Gao X, Peng M. Quantitative Intracerebral Iodine Extravasation in Risk Stratification for Intracranial Hemorrhage in Patients with Acute Ischemic Stroke. AJNR Am J Neuroradiol. 2022 Nov;43(11):1589-1596. doi: 10.3174/ajnr.A7671.
13. Bernsen MLE, Veendrick PB, Martens JM, Pijl MEJ, Hofmeijer J, van Gorp MJ. Initial experience with dual-layer detector spectral CT for diagnosis of blood or contrast after endovascular treatment for ischemic stroke. Neuroradiology. 2022 Jan;64(1):69-76. doi: 10.1007/s00234-021-02736-5.
14. Stanton M, Sparti G. Use of dual-energy computed tomography post endovascular treatment of cerebral aneurysm. Surg Neurol Int. 2021 May 17;12:225. doi: 10.25259/SNI_41_2021.
15. Yen HH, Chien C, Lee IH, Chen SP, Luo CB, Lin CJ, Chen ST, Yuan WH, Kuo Y, Wu CH, Chang FC. Application of Flat-Panel Volume Computed Tomography to Evaluate Cerebral Hemorrhage After Mechanical Thrombectomy of Acute Embolic Stroke of the Anterior Circulation. J Comput Assist Tomogr. 2021 Nov-Dec 01;45(6):919-925. doi: 10.1097/RCT.0000000000001203.
16. Yedavalli V, Sammet S. Contrast Extravasation versus Hemorrhage after Thrombectomy in Patients with Acute Stroke. J Neuroimaging. 2017 Nov;27(6):570-576. doi: 10.1111/jon.12446.
17. Cho WH, Choi HJ, Nam KH, Lee JI. Contrast Extravasation on Computed Tomography Angiography Imitating a Basilar Artery Trunk Aneurysm in Subsequent Conventional Angiogram-Negative Subarachnoid Hemorrhage: Report of Two Cases with Different Clinical Courses. J Cerebrovasc Endovasc Neurosurg. 2015 Dec;17(4):324-30. doi: 10.7461/jcen.2015.17.4.324.
18. Wu TC, Tsui YK, Chen TY, Lin CJ, Wu TC, Tzeng WS. Rebleeding of aneurysmal subarachnoid hemorrhage in computed tomography angiography: risk factor, rebleeding pattern, and outcome analysis. J Comput Assist Tomogr. 2012 Jan-Feb;36(1):103-8. doi: 10.1097/RCT.0b013e31823f2e57.

**Lack of reference standard verification (n=13):**

1. Brockmann C, Scharf J, Nölte IS, Seiz M, Groden C, Brockmann MA. Dual-energy CT after peri-interventional subarachnoid haemorrhage: a feasibility study. Clin Neuroradiol. 2010 Dec;20(4):231-5. doi: 10.1007/s00062-010-0036-3.
2. Luo J, Zhao X, Xiao M, Wei L, Zhu Z, Li B, Ji Z, Wu Y, Lin Z, Pan S, Huang K. Clearance rate of contrast extravasation after endovascular therapy is associated with functional outcome and mediated by cerebral edema. J Cereb Blood Flow Metab. 2025 Jan;45(1):66-76. doi: 10.1177/0271678X241275763.
3. Qiu T, Feng H, Shi Q, Fu S, Deng X, Chen M, Li H, Zhang Z, Xu X, Xiao H, Wang Z, Yu X, Tang J, Dai X. Dual-energy Computed Tomography (DECT) predicts the efficacy of contrast medium extravasation and secondary cerebral hemorrhage after stent thrombectomy in acute ischemic cerebral infarction. Biotechnol Genet Eng Rev. 2024 Apr;40(1):202-216. doi: 10.1080/02648725.2023.2183311.
4. Ito H, Nakamura Y, Togami Y, Onishi S, Nakao S, Ogura H, Oda J. Relationship between extravascular leakage and clinical outcome on computed tomography of isolated traumatic brain injury. Acute Med Surg. 2024 Feb 20;11(1):e931. doi: 10.1002/ams2.931.
5. Hoche C, Henderson A, Ifergan H, Gaudron M, Magni C, Maldonado I, Cottier JP, Pasi M, Boulouis G, Cohen C. Determinants and Clinical Relevance of Iodine Contrast Extravasation after Endovascular Thrombectomy: A Dual-Energy CT Study. AJNR Am J Neuroradiol. 2023 Dec 29;45(1):30-36. doi: 10.3174/ajnr.A8081.
6. Ho SK, Lee JK, Lai YJ, Lin TC, Liu CW. Differentiating contrast staining after acute ischemic stroke from hemorrhagic transformation during emergency evaluation. Am J Emerg Med. 2016 Nov;34(11):2255.e5-2255.e6. doi: 10.1016/j.ajem.2016.05.035.
7. Brouwers HB, Battey TW, Musial HH, Ciura VA, Falcone GJ, Ayres AM, Vashkevich A, Schwab K, Viswanathan A, Anderson CD, Greenberg SM, Pomerantz SR, Ortiz CJ, Goldstein JN, Gonzalez RG, Rosand J, Romero JM. Rate of Contrast Extravasation on Computed Tomographic Angiography Predicts Hematoma Expansion and Mortality in Primary Intracerebral Hemorrhage. Stroke. 2015 Sep;46(9):2498-503. doi: 10.1161/STROKEAHA.115.009659.
8. Rosa Júnior M, Rocha AJ, Saade N, Maia Júnior AC, Gagliardi RJ. Active extravasation of contrast within the hemorrhage (spot sign): a multidetector computed tomography finding that predicts growth and a worse prognosis in non-traumatic intracerebral hemorrhage. Arq Neuropsiquiatr. 2013 Oct;71(10):791-7. doi: 10.1590/0004-282X20130124.
9. Won SY, Schlunk F, Dinkel J, Karatas H, Leung W, Hayakawa K, Lauer A, Steinmetz H, Lo EH, Foerch C, Gupta R. Imaging of contrast medium extravasation in anticoagulation-associated intracerebral hemorrhage with dual-energy computed tomography. Stroke. 2013 Oct;44(10):2883-90. doi: 10.1161/STROKEAHA.113.001224.
10. Suzuki K, Ueno E, Kasuya H. Origin of sylvian hematoma in patients with subarachnoid hemorrhage: findings of extravasation on multiphase contrast-enhanced computed tomography. World Neurosurg. 2014 Dec;82(6):e747-51. doi: 10.1016/j.wneu.2013.02.014.
11. Li N, Wang Y, Wang W, Ma L, Xue J, Weissenborn K, Dengler R, Worthmann H, Wang DZ, Gao P, Liu L, Wang Y, Zhao X. Contrast extravasation on computed tomography angiography predicts clinical outcome in primary intracerebral hemorrhage: a prospective study of 139 cases. Stroke. 2011 Dec;42(12):3441-6. doi: 10.1161/STROKEAHA.111.623405. E
12. Kumar G, Soni CR, Sahota PK. Transient CT hyperattenuation after Merci clot retrieval and intraarterial thrombolysis in acute stroke mimicking subarachnoid hemorrhage. J Vasc Interv Radiol. 2010 Feb;21(2):281-4. doi: 10.1016/j.jvir.2009.10.022.
13. Murai Y, Ikeda Y, Teramoto A, Goldstein JN, Greenberg SM, Smith EE, Lev MH, Rosand J. Contrast extravasation on CT angiography predicts hematoma expansion in intracerebral hemorrhage. Neurology. 2007 Aug 7;69(6):617; author reply 617. doi: 10.1212/01.wnl.0000278894.44311.0b.

**Inclusion of other disease conditions (n=21):**

1. Chen X, Xu J, Zhang S, Guo S, Wang H, Shang Y, Shen P, Ye J, Geng Y. Modified Alberta Stroke Program Early CT Score (ASPECTS) of Contrast Extravasation on Dual-Energy CT Predicts Haemorrhagic Transformation and Poor Outcome After Endovascular Thrombectomy. Ther Clin Risk Manag. 2025 Dec 1;21:1603-1614. doi: 10.2147/TCRM.S522244.
2. Gunda B, Böjti P, Takács T, Zhubi E, Bereczki D, Varga A, Kozák LR. Spontaneous intracerebral hemorrhage during computed tomography scanning-assessment of hyperacute hematoma growth. Geroscience. 2025 May 10. doi: 10.1007/s11357-025-01696-5.
3. Abdulazim A, Rubbert C, Reichelt D, Mathys C, Turowski B, Steiger HJ, Hänggi D, Etminan N. Dual- versus Single-Energy CT-Angiography Imaging for Patients Undergoing Intracranial Aneurysm Repair. Cerebrovasc Dis. 2017;43(5-6):272-282. doi: 10.1159/000464356.
4. Scholtz JE, Wichmann JL, Bennett DW, Leithner D, Bauer RW, Vogl TJ, Bodelle B. Detecting Intracranial Hemorrhage Using Automatic Tube Current Modulation With Advanced Modeled Iterative Reconstruction in Unenhanced Head Single- and Dual-Energy Dual-Source CT. AJR Am J Roentgenol. 2017 May;208(5):1089-1096. doi: 10.2214/AJR.16.17171.
5. Bonatti M, Lombardo F, Zamboni GA, Pernter P, Pozzi Mucelli R, Bonatti G. Dual-energy CT of the brain: Comparison between DECT angiography-derived virtual unenhanced images and true unenhanced images in the detection of intracranial haemorrhage. Eur Radiol. 2017 Jul;27(7):2690-2697. doi: 10.1007/s00330-016-4658-y.
6. Wang D, Zhang Q, Hu H, Zhang W, Chen R, Zee CS, Yu R. Optimal Contrast of Cerebral Dual-Energy Computed Tomography Angiography in Patients With Spontaneous Subarachnoid Hemorrhage. J Comput Assist Tomogr. 2016 Jan-Feb;40(1):48-52. doi: 10.1097/RCT.0000000000000336.
7. Gariani J, Cuvinciuc V, Courvoisier D, Krauss B, Mendes Pereira V, Sztajzel R, Lovblad KO, Vargas MI. Diagnosis of acute ischemia using dual energy CT after mechanical thrombectomy. J Neurointerv Surg. 2016 Oct;8(10):996-1000. doi: 10.1136/neurintsurg-2015-011988.
8. Zhang LJ, Wu SY, Poon CS, Zhao YE, Chai X, Zhou CS, Lu GM. Automatic bone removal dual-energy CT angiography for the evaluation of intracranial aneurysms. J Comput Assist Tomogr. 2010 Nov-Dec;34(6):816-24. doi: 10.1097/RCT.0b013e3181eff93c.
9. Kreye MA, Schulze Westhoff M, Grosse GM, Renne J, Pirayesh A, Storti B, Bersano A, Weissenborn K, Krauss JK, Hartmann C, Gabriel MM. The Simplified Edinburgh Criteria in Clinical Practice: A CT-Neuropathology Accuracy Study for Diagnosis of Cerebral Amyloid Angiopathy. Neurology. 2025 Dec 9;105(11):e214349. doi: 10.1212/WNL.0000000000214349.
10. Shen Y, Ye T. Dual-Energy Computed Tomography (DECT) for Diagnosing Contrast-Induced Encephalopathy (CIE) Mimicking Intracranial Hemorrhage (ICH): A Rare Case. Diagnostics (Basel). 2025 Sep 23;15(19):2426. doi: 10.3390/diagnostics15192426.
11. Gulko E, Lerman S, Spirollari E, Zeller S, Mureb M, Wainwright J, Oishi A, Kouyoumdjian A, Clare K, Rozenshtein A, Al-Mufti F, Gandhi C, Mehta H. The delayed spectral sign in Post-transfer CT imaging: an imaging marker of stroke severity and hemorrhagic risk. Neuroradiology. 2025 Sep;67(9):2349-2356. doi: 10.1007/s00234-025-03718-7.
12. Vedicherla SV, Foo AS, Sharma VK, Ting EY, Sein Lwin, Chou N, Yeo TT. The "Blush" Sign on Computed Tomography Angiography is an Independent Predictor of Hematoma Progression in Primary Hypertensive Hemorrhage. J Stroke Cerebrovasc Dis. 2018 Jul;27(7):1878-1884. doi: 10.1016/j.jstrokecerebrovasdis.2018.02.018.
13. Fu F, Sui B, Liu L, Su Y, Sun S, Li Y. Quantitative assessment of local perfusion change in acute intracerebral hemorrhage areas with and without "dynamic spot sign" using CT perfusion imaging. Acta Radiol. 2019 Mar;60(3):367-373. doi: 10.1177/0284185118780893.
14. Suzuki K, Kurashima A, Abe K, Ishikawa T, Yamaguchi K, Kawamata T, Yaguchi A, Sakai S. Dual-Phase Computed Tomography Angiography Enhances Detection of Contrast Extravasation in Subarachnoid Hemorrhage. World Neurosurg. 2020 Feb;134:e237-e242. doi: 10.1016/j.wneu.2019.10.046.
15. Almqvist H, Almqvist NS, Holmin S, Mazya MV. Dual-Energy CT Follow-Up After Stroke Thrombolysis Alters Assessment of Hemorrhagic Complications. Front Neurol. 2020 May 19;11:357. doi: 10.3389/fneur.2020.00357.
16. Portela de Oliveira E, Chakraborty S, Patel M, Finitsis S, Iancu D. Value of high-density sign on CT images after mechanical thrombectomy for large vessel occlusion in predicting hemorrhage and unfavorable outcome. Neuroradiol J. 2021 Apr;34(2):120-127. doi: 10.1177/1971400920975259.
17. Rosa Júnior M, da Rocha AJ, Maia Júnior AC, Saade N, Gagliardi RJ. The active extravasation of contrast (spot sign) depicted on multidetector computed tomography angiography might predict structural vascular etiology and mortality in secondary intracranial hemorrhage. J Comput Assist Tomogr. 2015 Mar-Apr;39(2):217-21. doi: 10.1097/RCT.0000000000000182.
18. Hsu CC, Kwan GN, Singh D, Pratap J, Watkins TW. Principles and Clinical Application of Dual-energy Computed Tomography in the Evaluation of Cerebrovascular Disease. J Clin Imaging Sci. 2016 Jun 29;6:27. doi: 10.4103/2156-7514.185003.
19. Wu TC, Chen TY, Shiue YL, Chen JH, Hsieh TJ, Ko CC, Lin CP. Added value of delayed computed tomography angiography in primary intracranial hemorrhage and hematoma size for predicting spot sign. Acta Radiol. 2018 Apr;59(4):485-490. doi: 10.1177/0284185117718401.
20. Dowlatshahi D, Wasserman JK, Momoli F, Petrcich W, Stotts G, Hogan M, Sharma M, Aviv RI, Demchuk AM, Chakraborty S; Ottawa Stroke Research Group. Evolution of computed tomography angiography spot sign is consistent with a site of active hemorrhage in acute intracerebral hemorrhage. Stroke. 2014 Jan;45(1):277-80. doi: 10.1161/STROKEAHA.113.003387.
21. Nakano S, Iseda T, Yoneyama T, Wakisaka S. Early CT signs in patients with acute middle cerebral artery occlusion: incidence of contrast staining and haemorrhagic transformations after intra-arterial reperfusion therapy. Clin Radiol. 2006 Feb;61(2):156-62. doi: 10.1016/j.crad.2005.08.016.
